# Use of Blood Lactate in Assessment of Manual Capture Techniques of Zoo-Housed Crocodilians

**DOI:** 10.3390/ani12030397

**Published:** 2022-02-08

**Authors:** Holly Grace Molinaro, Gen S. Anderson, Lauren Gruny, Emily S. Sperou, Darryl J. Heard

**Affiliations:** 1 Psychology Department, Arizona State University, Tempe, AZ 85281, USA; 2 St. Augustine Alligator Farm Zoological Park, St. Augustine, FL 32080, USA; GAnderson@alligatorfarm.com (G.S.A.); LaurenG@alligatorfarm.com (L.G.); 3 Biology Department, Baylor University, Waco, TX 76707, USA; Emily_Sperou1@baylor.edu; 4 Department of Comparative Diagnostic and Population Medicine, College of Veterinary Medicine, University of Florida, Gainesville, FL 32610, USA; heardd@ufl.edu

**Keywords:** reptile, welfare, captivity, wellbeing, physiology, restraint

## Abstract

**Simple Summary:**

This study aimed to clarify the relationship between manual capture techniques, blood lactate levels, and other varying factors in order to minimize physiological stress during manual capture and restraint events of zoo-housed crocodilians. While following the St. Augustine Alligator Farm Zoological Park’s capture and restraint protocol, 53 blood samples from ten crocodilian species were collected and analyzed for lactate. These measurements were then related to specific behavioral and extrinsic factors. We sought to define blood lactate as a new welfare marker for crocodilians in zoos. Based on our findings, we are able to recommend some best practices for manual capture methods for crocodilians.

**Abstract:**

Species-specific welfare indicators are important in promoting positive welfare for zoo animals. Reptiles are a notoriously understudied group in regards to behavior, welfare needs, and husbandry requirements. Using opportunistically obtained samples, we evaluated how blood lactate is affected by variation in manual capture and restraint in crocodilians. Lactate is an indicator of anerobic metabolism in reptiles. It offers a relatively simple and indirect way to assess physiological stress. Determining the best way to reduce struggling during capture and associated stress is of great importance to these species’ overall welfare. Blood samples (*N* = 53) were collected from 10 different species of crocodilians. It was found that age class was a significant predictor variable of lactate levels after capture, and longer handling time seemed to cause an increase in lactate. Finally, draining enclosure pools for a small number of the captures was associated with higher lactate levels compared to other capture factors that were recorded. This study showed that welfare of captive crocodilians could be improved by shortening the duration of physical restraint events when possible. Developing objective measures of welfare and establishing baseline recommendations for care and handling of crocodilians will ultimately promote and improve their wellbeing, along with that of other understudied reptiles in zoos.

## 1. Introduction

Despite increased studies in recent years, there is still relatively little known about the welfare indicators of reptiles housed in zoos and aquariums (henceforth zoos). Captive studies of behavior and welfare are more common in mammals compared to other vertebrates, with little attention given to the understanding of reptiles’ natural behaviors because they are thought to have a simpler lifestyle [1,2,3,4]. Specifically for zoo-housed crocodilians (alligators, crocodiles, caimans, and gharials), there are numerous behavioral and extrinsic factors involved in improving their overall welfare [5], yet little has been empirically studied from a zoo perspective. Most research is from crocodile farms where the primary focus is to promote quality hide and meat production [6]. Therefore, there is a current need for more research on the welfare requirements of zoo-housed crocodilians.

An important aspect of crocodilian welfare is the capture and/or restraint of individuals for various husbandry needs including but not limited to: transportation, medical examinations, forming appropriate reproductive pairings, and environmental concerns [7]. Safe, effective capture and restraint protocols are an important aspect to the care and welfare of zoo crocodilians due to the potential danger to themselves and personnel. While there are studies on the welfare and health concerns of other animals during capture and restraint events, including even the potential of mortality caused by elongated capture [8,9,10], this area of zoo-housed crocodilian care is understudied. If the capture is not performed appropriately, severe health issues can occur due to cascading physiological effects of increased stress [11]. Consequently, by measuring physiological stress as an indicator of welfare [11,12,13], we can assess capture and restraint methods on crocodilians to thus improve their overall wellbeing in zoos.

Stress is a natural biological response to an extrinsic or intrinsic event that includes behavioral and physiological changes, including neuroendocrine and immune responses [11]. Hormone monitoring is one of the most common ways to measure physiological stress in animals, with corticosterone being the main stress hormone indicator in reptiles [14]. A study found higher values of corticosterone in American alligators (*Alligator mississippiensis*) housed at high stocking densities and concluded this hormone was effective in stress regulation of alligators [15]. Similarly, corticosterone has also been shown to increase in response to manual capture in freshwater crocodiles (*Crocodylus johnstoni*) and American alligators [16,17].

In reptiles, corticosterone is correlated with blood lactate levels [18,19]. This measurement, thus, is another way to indirectly assess physiological stress [18,20,21,22]. Lactate is a by-product of anaerobic metabolism using stored glycogen within muscle cells [23]. In crocodiles, lactate is produced during vigorous, though brief, bursts of energy, such as when they explosively leap out at their prey, react powerfully in a territorial outburst, or when startled [20,24]. In small amounts, it is not harmful, but does hinder general mobility until metabolized [25]. Prolonged extreme anaerobic metabolism results in marked lactic acidemia that can result in death primarily from cardiac dysfunction [26]. Unlike mammals, crocodilians and reptiles in general lack the ability to rapidly metabolize and correct this acidemia [27]. Previous studies have exercised saltwater crocodiles (*Crocodylus porosus*) of various sizes to extreme exhaustion with lactate values reaching life-threatening levels [26,28]. A major aim of crocodilian capture and restraint should be to minimize lactate production, thereby maintaining physiological stability and indirectly reducing stress in the animal.

While limited, there are studies that have used lactate as a measure of welfare in other animals, including pigs raised for meat production [29,30], deer in a wildlife rescue center [31], and the effect of capture in wild tortoises, turtles, and snakes [18,32,33]. One study on zoo-housed American flamingoes (*Phoenicopterus ruber*) used lactate to evaluate the effects of capture time and difficulty, finding that lactate levels significantly increased with time of capture but not with difficulty of capture [34]. Specifically for crocodilians in farmed settings, blood lactate has been used to assess physiological stress regarding capture and restraint methods [22]. There are several ways to capture and restrain crocodilians, and different techniques can increase or reduce stress for an individual [35]. Sedation using the drug midazolam has been shown to reduce physiological stress as lactate levels increased 5-fold with manual capture compared to sedation in saltwater crocodiles [36]. However, the drug is expensive, requires large volumes in medium to large crocodilians, and may take a long time (two to four days) for an individual to fully recover from its effects [36]. Electro-stunning has been used to temporarily restrain saltwater crocodiles, resulting in an increase of plasma lactate concentrations, though not nearly as significant as the 12-fold increase by manual capture [37]. Another study found lactate levels were significantly lower in Nile crocodiles (*Crocodylus niloticus*) when electro-stunned for capture compared to noosing [22]. These results indicate the importance of determining appropriate methods to conduct and then assess capture and restraint events without incurring additional stress in crocodilians.

Free-living crocodilians are exposed to many stressors that can be magnified in captivity. Stress will vary with companions, seasonal changes, enclosure size and design, reproduction, and capture events [17,38,39]. Since lactate is an indicator of more extensive anaerobic demands, such as those unavoidable during manual capture, it can be a way to indirectly measure physiological stress in crocodilians. Portable lactate meters are a cost-effective, convenient device which requires only a small amount of blood once the crocodilian is safely restrained and provides an instant result. To our knowledge, there is no research on baseline lactate levels for zoo-housed crocodilians, or any reference lactate levels indicative of appropriate capture techniques.

The main objective of this study was to understand the effects of manual capture and restraint methods on opportunistically obtained blood lactate samples taken during routine translocation, medical, and other husbandry events in a zoological park. We aimed to determine lactate levels of numerous individuals and species of crocodilians during capture while incorporating behavioral and other extrinsic factors. Ultimately, our goal was to see if lactate levels increased with capture time in order to avoid the extreme and harmful effects of physiological stress while promoting a swift resumption of normal behavior. Finally, this study was intended to improve best practices for capture and restraint of Crocodilia and be a basis for future studies moving forward.

## 2. Materials and Methods

### 2.1. Study Sites and Animals

Blood was collected from 53 individuals of ten crocodilian species for lactate measurement between October 2018 and December 2019. The primary study site (*N* = 37) was the St. Augustine Alligator Farm Zoological Park (SAAF) in St. Augustine, Florida, USA, which is an accredited member of the Association of Zoos and Aquariums (AZA). These animals were maintained in enclosures varying from 850 to 10,000 square feet. Some enclosures were on-display to the public while others were behind the scenes. Additionally, 16 individuals were studied from Gatorama Inc. (Palmdale, FL, USA). All of these animals lived in a pond that was roughly two acres in size and on-display near the facility entrance.

### 2.2. Manual Capture and Restraint Protocol

**Figure 1:**
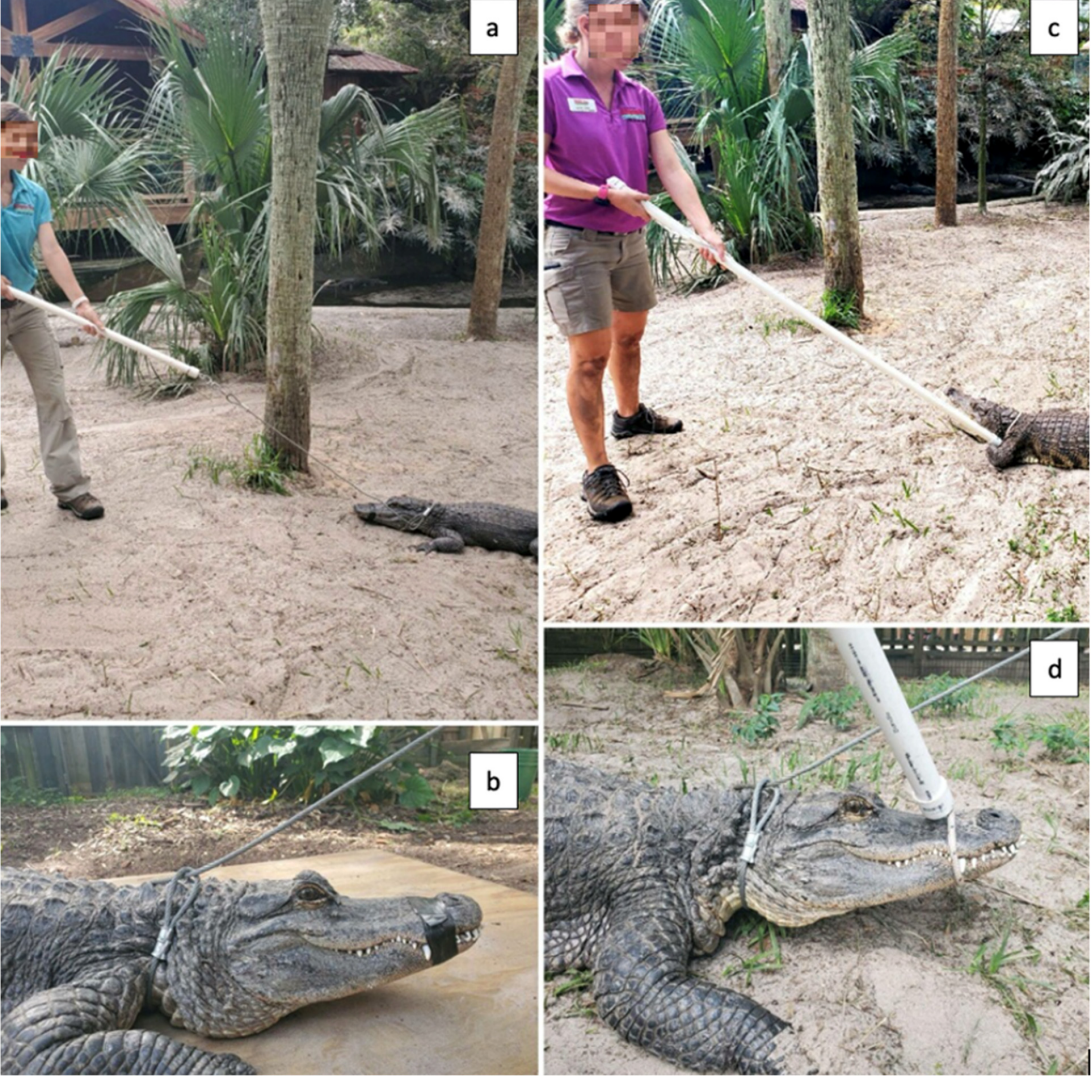
Usage of mouth pole and catch pole restraint equipment. (**a**) Catch pole with cable pulled taut around neck, typically used for crocodilians >6 ft in length. (**b**) Catch pole cable in proper position in front of legs and behind jowls. (**c**) A lightweight, easy to maneuver mouth pole is used for smaller crocodilian captures. (**d**) Mouth poles are also used to close jaws prior to securing with electrical tape.

The same manual capture and restraint protocol was used at all locations. Different capture techniques were used, however, due to animal size variation. All followed SAAF’s Capture and Restraint Crocodilian Policy and Procedures manual (see Supplementary Materials) and were led by fully trained staff members. The goal for all captures was to have the animal caught as safely and quickly as possible to avoid prolonged anaerobic activity [28]. Most capture equipment used at the primary study location was manufactured in-house [35]. For smaller animals, a mouth pole (¼ inch diameter rope that is attached through 1-inch PVC pipe) was used around the neck to initially restrain the animal (Figure 1c,d). Once the animal was pulled into a safe area with the pole, another mouth pole was used to circle the animal’s mouth and pull it shut. A staff member would then carefully tape the animal’s mouth shut with PVC electrical tape. Once this had been accomplished, one to two staff members applied weight on top of the animal to minimize movement when necessary.

With larger animals, there were two manual capture techniques used. The most common involved a catch pole (PVC pipe equipped with a looped, sliding cable that can cinch down) instead of the mouth pole around the neck (Figure 1a,b). However, for the largest individuals, a heavy rope was used around the animal’s neck and at least one front leg. The animal was then physically pulled to a safer area. Both the catch pole and heavy rope are sturdier options when capturing larger crocodilians. The same techniques were then applied as described for smaller animals. Animals that were caught either for enclosure moves or for medical treatment were often restrained on a backboard with straps to minimize struggling and ease transfer.

### 2.3. Data Collection

All blood samples were collected opportunistically as part of already scheduled veterinary evaluations, other research studies, routine body examinations including weight and growth measurements, and/or husbandry moves. Two of the *A. mississippiensis* with medical conditions included in this study were chronic in nature, but did not compromise their activity levels. The Gatorama individuals have historically rarely had hands on their animals. Translocation of crocodilians is not uncommon at SAAF, but seldom more than once a year and typically far less often when of adult size.

Blood was collected by trained staff members once the animal was safely restrained with a heparinized syringe and needle (22-gauge, length varied between 1–2 depending on the size of the crocodilian) from the post-occipital spinal vein [40]. This blood draw location was selected for ease and rapidity of collection [28]. The one exception was a sample collected from the tail (ventral coccygeal vein) of an adult Indian gharial (*Gavialis gangeticus*) behaviorally trained for a voluntary blood draw. A commercial blood lactate analyzer (Lactate Plus Meter, Nova Biomedical, Waltham, MA, USA) was used to measure one drop of the blood sample immediately after collection. Sex, age class (non-adult or adult), and weight were recorded (when possible) with each capture along with the date, time duration of the capture to blood collection, and any unique capture factors that occurred during the procedure. The time of capture onset began once staff entered an enclosure with capture equipment and the crocodilian of interest was visible. Capture time stopped after safe restraint, but prior to release, once blood was collected from the animal. Since this was an opportunistically obtained data set, some descriptive values were unable to be recorded due to extraneous and varying influences on the procedure in a captive setting. Lactate levels were documented into the medical component of the global zoo database ZIMS (Zoological Information Management System) to increase the sample size of reference values for other facilities.

### 2.4. Data Analysis

JMP Pro 15.0.0 (SAS Institute Inc., Cary, NC, USA) was used for all data analyses. Statistical significance was assessed at *p* < 0.05 and degrees of freedom for F statistics were calculated using the Kenward–Rogers method. Data were independent with approximate normal distribution and equal variances. Because these are captive animals and sampling was done the same way at both SAAF and Gatorama in addition to location not being of experimental interest, all data was combined and location was excluded from the analysis. Due to uneven sample sizes across species, this variable was also excluded from data analysis. However, reference data on lactate concentrations are presented for each species group in Table 1.

#### 2.4.1. Predictors of Lactate

A linear regression model was used to identify which predictor variables were of relative importance for lactate, with lactate as the response variable and age class, sex, weight, and time from capture to blood collection set as fixed effects. We used a backward stepwise approach to eliminate variables one-by-one based on significance level. An ANOVA test was then used to analyze significant differences within groups. Post hoc comparisons were performed using Student’s *t*-tests. Data were expressed as ± standard deviation of the mean.

#### 2.4.2. Relationship between Lactate, Time from Capture to Blood Collection, and Weight

Additionally, variable relationships between time of capture to blood collection, weight, and lactate were evaluated. A linear regression was used to assess the association between lactate and time from capture to blood collection, with lactate as the response variable and time from capture to blood collection as the fixed effect. Next, a linear regression was used to assess the association between lactate and weight, with lactate as the response variable and weight as the fixed effect. Lastly, a linear regression was used to look at the relationship between weight and time from capture to blood collection.

#### 2.4.3. Visual Differences in Lactate with Capture Factors

A boxplot was used to visually assess differences between a subset of the data in which there were unique capture factors and their associated lactate levels (*N* = 41). Due to uneven sampling between groups and low sample sizes, no statistical tests were run. The factors were: drained pool (*n* = 3) (in which the pool had to be drained for the capture to be completed), active (*n* = 8) (in which the animal was highly behaviorally active during the capture process), above 10 min (*n* = 11) (in which the time the capture began to blood draw took over 10 min to complete), and quick capture (*n* = 19) (in which the time the capture began to blood draw took 10 min or less).

## 3. Results

Within our dataset, we removed any time from capture to blood collection outlier when it was more than two standard deviations above the mean (18.17 min). Only one outlier was removed from the data set. Within our data set (*N* = 53), there were 10 different species, with 17 known females, 21 known males, and 15 unknown sexes. The mean weight for the males was 93.20 ± 81.67 kg, the mean weight for the females was 93.40 ± 63.44 kg, and the mean weight for the unknown group was 128.77 ± 38.55 kg. Mean lactate concentration was 12.99 ± 5.04 mmol/L. The lowest lactate value after handling and capture was 1.20 mmol/L, which was the one voluntary blood draw from an Indian gharial described above in methods. The next lowest lactate value was 3.00 mmol/L and the highest was 21.00 mmol/L. The average time from capture to blood collection was 12 ± 9.21 min. Sex, weight, and mean lactate concentrations for each species are presented in Table 1.

### 3.1. Predictors of Lactate

From our stepwise linear regression procedure, we found sex (F_2152_._0 =_ 3.89, *p* = 0.02) and age class (F_1111_._35_ = 5.70, *p* = 0.02) to be the only significant predictors of lactate. Weight and time of capture were not significant and one by one removed from the model. Further post-hoc analysis showed there was no significant difference in lactate concentrations between the known males and females with average lactate levels being 9.69 mmol/L and 10.49 mmol/L, respectively. However, the unknown group had significantly higher lactate values than males and females (*p* < 0.05), with an average of 13.94 mmol/L. Additionally, there was a significant difference between age class (*p* < 0.05) with non-adults having lower lactate values compared to adults, with an average of 8.51 mmol/L and 13.90 mmol/L, respectively (Table 2).

### 3.2. Relationship between Time from Capture to Blood Collection and Lactate

**Figure 2:**
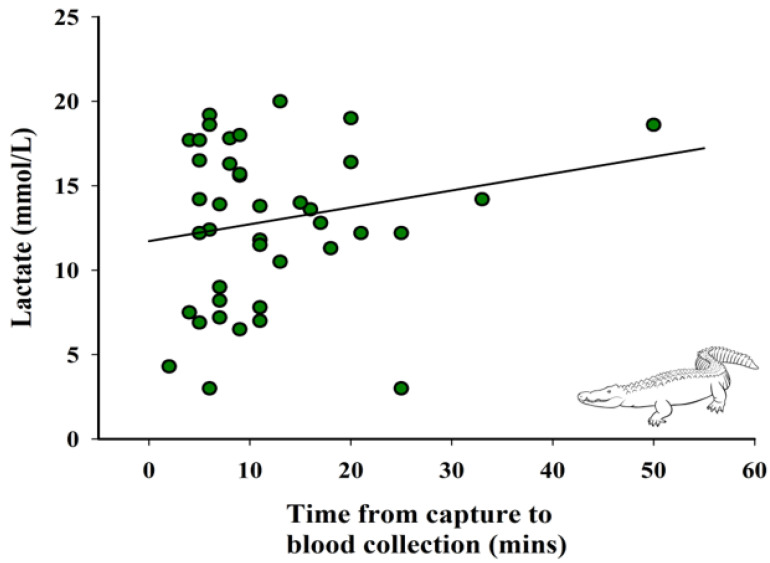
Relationship between lactate concentrations and time from capture to blood collection (*n* = 40). Fitted line is based on the linear regression parameter estimates, R^2^ = 0.02, *p* > 0.05.

When visually assessing, lactate values increase with time of capture (Figure 2). However, there was no significant relationship between time from capture to blood collection and lactate concentrations (*p* > 0.05).

### 3.3. Relationship between Weight and Lactate

**Figure 3:**
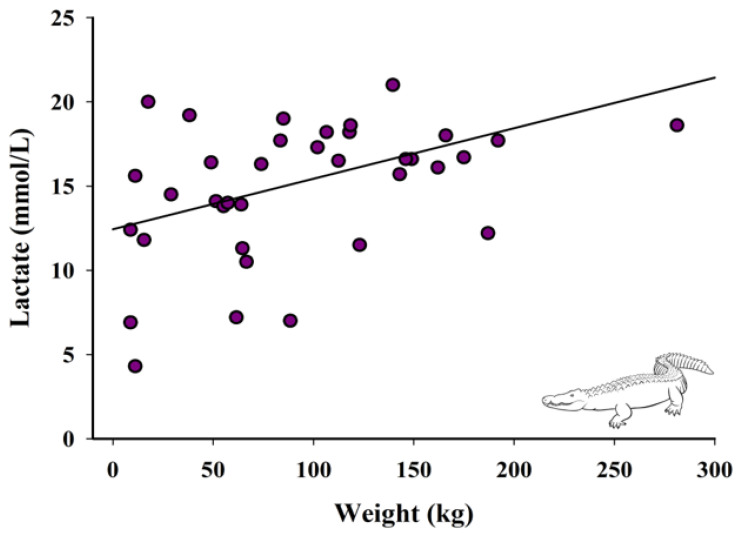
Relationship between weight and lactate concentrations of crocodilians (*n* = 36). Fitted line is based on the linear regression parameter estimates, R^2^ = 0.17, *p* = 0.01.

There was a significant positive relationship between weight of the crocodilian and lactate values (F_1,96_ = 6.94, *p* = 0.01; Figure 3).

### 3.4. Relationship between Weight and Time from Capture to Blood Collection

**Figure 4:**
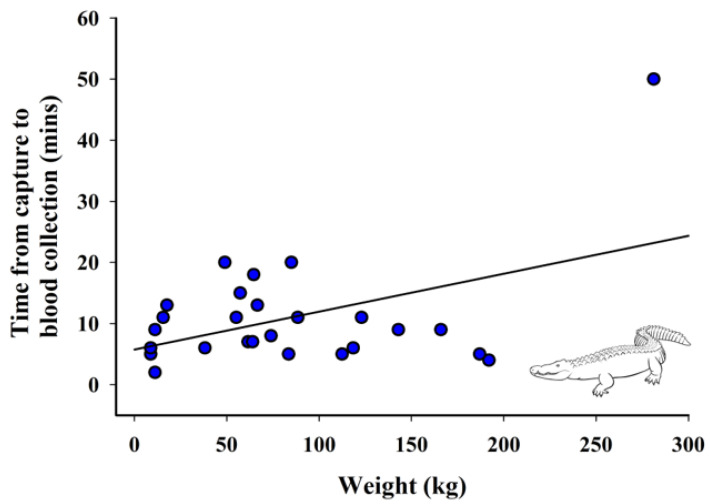
Relationship between weight and time from capture to blood collection of crocodilians (*n* = 26). Fitted line is based on the linear regression parameter estimates, R^2^ = 0.20, *p* = 0.02.

There was a significant positive relationship between weight of the crocodilian and time from capture to blood collection (F_1440_._15_ = 6.14, *p* = 0.02; Figure 4).

### 3.5. Visual Differences in Lactate with Capture Factors

**Figure 5:**
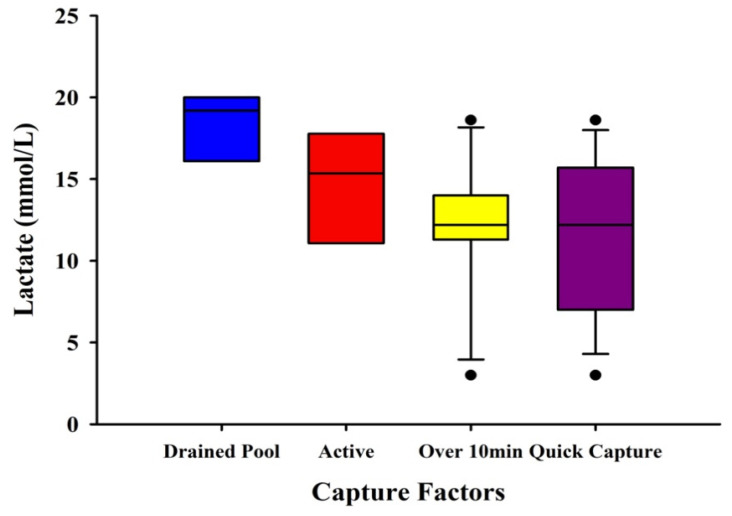
Boxplot of lactate measurements from multiple species of crocodilians with different capture factors. The factors were: drained pool (*n* = 3) (in which the pool had to be drained for the capture to be completed), active (*n* = 8) (in which the animal was highly behaviorally active during the capture process), above 10 min (*n* = 11) (in which the time the capture began to blood draw took over 10 min to complete), and quick capture (*n* = 19) (in which the time the capture began to blood draw took 10 min or less). Whiskers show minimum and maximum values of the capture factors with enough data.

The boxplot shows visual differences between the different capture factors (Figure 5). Drained pool had the highest mean lactate levels of 18.43 mmol/L, while quick capture had the lowest mean lactate levels of 11.52 mmol/L.

## 4. Discussion

This study used blood lactate as an indicator of physiological stress and welfare during manual capture of zoo-housed crocodilians. The magnitude of lactate elevation was related to age class and it seems the duration of capture also appears to influence lactate values. There were also effects of weight on lactate and on time of capture. In addition, there were differences in lactate levels with the unique capture factors. Reference values were determined for elevated lactate concentrations following a manual capture event for 10 different species of crocodilians. With blood lactate samples collected in a number of different scenarios, this study ultimately proposes ways of improving manual capture methods for captive crocodilians, as well as provides a starting point for further research.

Non-adult crocodilians had significantly lower lactate levels after capture than adults. Therefore, the age of the crocodilian itself is a significant predictor of lactate levels after capture. It is important to note that unless a hatch year is known, age estimates can be difficult to determine in crocodilians. Growth rates are influenced by factors such as climate, length of seasons, available nutrition, gender, and social dynamics [41]. When a hatch date was unknown in our sample, age class was determined using hatch estimates, weight and length measurements, and acquisition into the zoo’s collection.

In other reptiles, including the Eastern box turtle (*Terrapene carolina carolina*) and the gopher tortoise (*Gopherus polyphemus*), age class (immature/juvenile or adult) was not found to have an effect on lactate after a capture event [32,42]. In other studies that assessed lactate in crocodilians, age was not mentioned [22,36,43]. Overall, our results showcase how age class of crocodilians can affect the overall stress of a capture event, as they are going through different physiological stages with age [44]. Based off our model, sex was also of relative importance when predicting lactate; however, the known sex groups (males and females) did not significantly differ in average lactate values, while our unknown sex group of crocodilians did differ in average lactate values. We believe a more complete data set is needed to clarify the relationship between sex and lactate levels in captive crocodilians.

Visual assessment of the data suggests the longer the capture time, the higher the lactate level. The length of capture time in our study varied due to a variety of different reasons, including time spent locating the animal in a group setting, difficulty in restraining both jaws in some species, draining the pool in order to uncover the individual, working with species that readily roll to avoid capture, or even the capture of an adjacent animal first. In group or adjacent settings, once one individual was caught and restrained, others typically hid in harder to access areas, or would stay submerged in deeper water. Therefore, conspecifics in captive settings could affect varying capture factors and could potentially elevate lactate values, but further research is needed to confirm this. While the association of time of capture and lactate was not statistically significant, Figure 2 does showcase a strong trend, indicating the value of this relationship and a convincing case for further study with a larger and more robust sample size.

Capture time was also hindered by older enclosure structures such as small doorframes, narrow corridors, and tight access points (personal communication, Gen Anderson 9/17/2021). SAAF has some enclosures, pools, and holding areas that were historically designed for basic functionality, not modern safe capture protocols. It is mandatory to evaluate enclosure size and complexity prior to the manual capture of crocodilians. While structures and furniture in the enclosure such as pools, bridges, plants, etc. are necessary for overall animal welfare, they can also hinder safe and quick capture attempts. Captive crocodilian enclosure design should be functional for both welfare needs and keeping manual capture and restraint methods as safe and short in time as possible.

Previous studies have indicated similar results in relation to time of capture and increased lactate levels. In zoo-housed animals, American flamingo lactate levels increased with capture time [34]. In reptiles, capturing and handling of wild gopher tortoises rapidly increased their lactate levels [18]. The same effect was found in Eastern box turtles [32] in addition to Eastern rat snakes and Eastern copperheads (*Pantherophis alleghaniensis*, *Agkistrodon contortrix*; [33]). Specifically in crocodilians, a lab-based study designed to exercise American alligators until exhaustion revealed elevated lactate levels over time [43]. It was found that four to six hours post, the alligators continued to have higher lactate levels compared to baseline (~5 mmol/L, 0 mmol/L baseline). These findings support that the best practices for capture events of crocodilians should be kept as short as possible when feasible.

Weight significantly impacted lactate levels after capture and affected the length of capture time. This could be because as crocodilians grew in size, so did the complexity of the capture, as SAAF staff noted that larger animals did pose more problems (personal communication, Gen Anderson 9/17/2021). Smaller animals under six feet in length could be captured safely and typically more quickly by two fully trained staff members. Animals that were over six feet ideally required three staff members for a capture. Any animal over nine feet would require a much larger team to be able to safely capture and restrain an animal. While weight is not the same as length, it is correlated [45,46].

A previous study indicated that size also had an effect on aerobic metabolism in that intensity, duration, and nature of activity changed with body size in saltwater crocodiles [47]. Conversely, another study found that both resting and active (after 2 min burst of activity) lactate levels were not affected by body size in four different species of reptiles (California legless lizard (*Anniella pulchra*), Eastern glass lizard (*Ophisaurus ventralis*), Checkerboard worm lizard (*Trogonophis wiegmanni*), and Butler’s garter snake (*Thamnophis butleri*)) [48]. However, that study investigated smaller sized reptiles compared to the much larger crocodilians in our study. In general, best practices of manual capture protocols should ensure that the weight of the crocodilian is considered when planning a capture event in order to have enough staff available to minimize elapsed time and ultimately prevent elevated lactate levels.

Finally, draining a pool to complete a capture resulted in considerably higher lactate levels compared to other capture factors. Crocodilians during this scenario took full advantage of their “mobile, semiaquatic, and cryptic nature” [24], effectively and efficiently evading staff. It is important to note that exhaustion in water can result in drowning as breathing overpowers normal submersion response [26], (although this did not occur during our study). Overall, avoiding this scenario in which the animal is at risk of drowning is crucial. Crocodilian captures can be quicker on land, but many times the onset of capture occurs when they are in the water. Zoo staff should ensure to note the time a capture specifically begins as determining the time that has passed in the moment can be difficult. If the capture is progressing slowly or the animal has been submerged for an extended period of time, staff should pause the capture event to reassess the situation.

We visually assessed differences in lactate levels after capture among the 10 different species sampled. While this makes interpretation limited, it does suggest the possibility of species-specific characteristics to consider during capture. For example, reclusive species, like the smooth-fronted caiman (*Paleosuchus trigonatus*), were difficult when trying to catch them in water. Species that are known for their agility were difficult to capture and restrain safely, such as the Cuban crocodile (*Crocodylus rhombifer*). Species like the Indian gharial with slender snouts had a tendency to have longer captures since there was concern over the animal injuring their jaws on concrete surfaces. In addition, while not sampled in our study, saltwater crocodiles are also prone to resistance and struggling during capture by rolling and repeatedly lashing their tail [37]. Understanding species-specific behaviors, along with individual characteristics, are of great importance before manual capture and restraint of any species, especially crocodilians.

Of note, one of the Indian gharial was a voluntary blood draw, as mentioned in the methods. This gharial had the lowest lactate values of all the individuals. This showcases the powerful effect that training and desensitization can have in alleviating physiological stress during routine procedures, thus increasing overall welfare. This anecdotal evidence further demonstrates that shorter handling time and voluntary blood draws results in lower lactate levels.

Resting lactate levels of farmed crocodiles have been established of 4.00 mmol/L [37] while life-threatening levels can reach 50.00 mmol/L [26,28]. In our study, our lowest values ranged from 1.20 to 3.00 mmol/L and our highest lactate level was 21.00 mmol/L. While we demonstrate a range of lactate levels after handling and manual capture, next steps should detail baseline resting lactate levels for zoo-housed crocodilians. This could be done through training and voluntary blood draws from the tail. Reptiles have the capacity to be trained through desensitization and conditioning [49] which can be a useful tool as mentioned above. More research is also necessary to set limits for capture times, minimize adverse extrinsic factors, and identify new capture techniques to reduce struggling.

In addition, we note that the Lactate Plus Meter has not been validated for any crocodilian species, but was developed as an aerobic conditioning tool for athletes. However, this device has been used for other reptiles [32,42,50] and has been validated for other vertebrate species [51,52]. Lastly, seasonality can have strong impacts on crocodilians’ behavior and physiology [53]. This can be associated with both the reproductive time frames and climate. Monitoring the season in which manual capture occurs in relation to lactate levels could yield additional promising results regarding best practices for this process. In adding our lactate levels to the international ZIMS database, we encountered no other baseline or analogous lactate levels for comparison. This highlights the importance of future research in this domain.

## 5. Conclusions

During this opportunistic preliminary study, we found that when assessing blood lactate levels in zoo-housed crocodilians after capture, age class was of relative importance when predicting lactate, lactate varied with duration of capture time, and draining the pool to complete the capture resulted in extremely elevated lactate levels. While this is the first study to assess lactate levels in zoo crocodilians, further research using a larger sample size and more controlled study design is warranted to determine more specific recommendations to ameliorate this stressor.

## Figures and Tables

**Table 1:** Sex, average weight, and mean ± SD lactate concentrations after capture for 10 different species.

Species	Sex	Average Weight (kg)	Lactate (mmol/L)
*A. mississippiensis*	F = 9 M = 10	F = 11.2 M = 281.22	10.02 ± 4.94 (*n* = 19)
*A. sinensis*	F = 1	F = 15.6	11.8 (*n* = 1)
*C. acutus*	M = 1 Unknown = 15	M = 166 U = 128.77	16.59 ± 2.4 (*n* = 16)
*C. halli*	M = 2	M = 59	12.3 ± 2.54 (*n* = 2)
*C. intermedius*	F = 1	F = 64.6	11.30 (*n* = 1)
*C. rhombifer*	F = 3 M = 1	M = 55.2 F = 69.13	10.5 ± 3.93 (*n* = 4)
*C. suchus*	F = 2 M = 2	M = 93.5 F = 39	16.8 ± 1.87 **(***n* = 4)
*G. gangeticus*	M = 2	M = 162	8.65 ± 10.53 (*n* = 2)
*P. trigonatus*	F = 1 M = 1	M = 38.2 F = 17.6	19.6 ± 0.56 (*n* = 2)
*T. schlegelii*	M = 2	M = 8.8	9.65 ± 3.88 (*n* = 2)

**Table 2:** Mean ± SD of lactate concentrations after capture with different age classes (*n* = 53). Post hoc Student’s *t*-test revealed significant differences (*p* = 0.002). Different superscripts (^A,B^) denote significant mean lactate differences between age class.

Age Class	Lactate (mmol/L)
Adult (*n* = 44)	13.90 ± 4.75 ^A^
Non-adult (*n* = 9)	8.51 ± 4.10 ^B^

## Data Availability

Data are available on request from the authors.

## Supplementary Materials

The following are available online at https://www.mdpi.com/article/10.3390/ani12030397/s1, St. Augustine Alligator Farm Zoological Park Capture and Restraint Crocodilian Policy and Procedures Manual.
