# Supplementary material for: Use of Blood Lactate in Assessment of Manual Capture Techniques of Zoo-Housed Crocodilians
Source: Animals (Basel). 2022 Feb 8;12(3):397. doi: 10.3390/ani12030397 (PMC8833426; doi:10.3390/ani12030397)
Supplement: Supplementary file 1 [file animals-12-00397-s001.zip › animals-1543049-supplementary.pdf]

Capture and Restraint Protocol

Crocodilian Policy and Procedures

St. Augustine Alligator Farm Zoological Park

General

Remember, Safety always comes first! There is nothing more important than the safety of the individuals working and visiting the St. Augustine Alligator Farm. Large crocodilians are extremely quick and powerful animals that should always be treated with the greatest respect. Complacency often leads to injury. Expect the unexpected! Proper, professional behavior must be exhibited and maintained at all times.

Materials

The different tools and materials used to capture crocodilians will depend on the size of the animal and the purpose of the capture. Materials that should be present at a crocodilian restraint are:

- PVC electrical tape (no less than 2 rolls at any time)
- Two bamboo poles
- One catch pole (pole equipped with a cable or wire rope sliding noose on one end)
- One towel for use as an eye cloth
- Two mouth poles (PVC Pole, capped on both ends with a rope roughly ¼ inch diameter fed through a 1 inch diameter pole, 3 - 5 feet long) Has a working noose on one end and a single rope on the other end for drawing the noose closed.
- Capture ropes (if large animal)
- Transponder reader (chips if needed)
- Measuring tape
- Pen and paper
- Two hooks for removal of rope (cattle show sticks work well)
- Knife or multi-tool

The capture and restraint of crocodiles and alligators is dangerous for even the most experienced handler. Those persons with little or no experience have an increased chance of accident or injury occurring. There are many capture techniques available, some being preferred over others. The technique decided on will depend on the number of personnel, equipment available, size of the animal(s), and the experience of the

personnel. Patience is very important when working with crocodilians. IF you are not succeeding in capturing an animal right away, discuss a new method, and/or try later.

The size of the animal(s) that are worked with are divided into different categories:

- Hatchlings- those that have recently hatched and have not reached their first birthday.
- Yearlings- an animal that has reached its first year.
- Juveniles (sub-adults)- animals between 2 and 4 feet long (depending on species)
- Sub-adult- typically between 4 feet in length (depending on species) and sexual maturity
- Adults- those animals that are of breeding age and/or size
- Large- those animals that are over nine feet in length

Hatchlings will usually be captured by hand by grasping the animal from above in the neck area. Use caution as even hatchlings will bite and have very sharp teeth.

Yearlings may also be captured by hand and/or small noose ropes around the neck area. Again, caution must be used as the animal may bite and at this size could cause a significant injury.

Juveniles and Sub-adults may possibly be captured by hand, but extreme caution must be used. Sub-adults of smaller species may also be captured using this method. Animals this size are very fast and strong. They may spin and bite quickly, and may inflict a severe wound. A noose rope applied over the head, around the neck, and pulled tight is a safe way to restrain an animal this size. If the animal is to be captured, maintaining a tight rope and approaching the animal from the rear, holding the tail to maintain the animal in a straight line will assist in control and give the animal little chance to spin. Reaching down low across the back and grabbing the animal at the neck will usually allow for control to be accomplished.

Never assume that the actions of one animal are common to all animals of that species. Crocodilians are individuals within a given species; some are aggressive while others may be quite passive. ***Always expect the unexpected, remember safety first.***

Adult crocodilians should be restrained by attaching a length of heavy rope around the neck of the animal and one front leg. Including the front leg minimizes risk of injury to the animal as it may fight, twist, and spin causing damage to the head, vertebrae, and neck area. On occasion these injuries could result in paralysis or in death. Once an adult crocodilian has been noosed, pull to an open area to avoid injury and increase safety.

Heavy cloth can be soaked and dropped over the eyes of a crocodilian to calm the animal and diminish any struggling. Be prepared to have to position the cloth several times as the animal may throw it off if not placed gently and appropriately.

A mouth pole (a rope of approximately ¼ inch in diameter attached to a PVC pole) is looped over the jaws and cinched to close the mouth. Assistance in the closing of the mouth may be required by placing the end of a pole under the bottom jaw as leverage. Push up carefully while pulling the mouth rope tight. Once the rope is in place and mouth secured, the jaws can then be taped.

Tape can also be added over a crocodilian's eyes at this time, but only rarely required. Taping the eye cloth on can be helpful when animal restraint time is longer for medical procedures. If it is necessary for the animal to be restrained for sexing or a blood draw, additional temporary resistance can be provided by allowing staff to sit along the crocodilian's back. Staff are not to sit comfortably, but perched ready to react while providing resistance. Another person can hold the crocodilians tail in order to minimize twisting. The person holding the tail will not be able to physically control the tail from swinging, but will be the first to know if the animal is tensing up and can communicate accordingly.

Animal Release

The release of captured or restrained animals can be just as dangerous as the capture. These risks can be minimal if the proper techniques are followed. Release by hand is limited to smaller animals by trained and authorized personnel only.

1. If possible, always carry the animal to the furthest most point away from the gates and or entry points of the enclosure.
2. Place the animal as close as possible to the water's edge. Use caution that the animal is not so close as to possibly move into the water while still restrained. This may result in an additional capture and more stress on personnel and animal.
3. Prior to final release, all unnecessary personnel must vacate the area and be on standby outside the enclosure out of exit pathways.
4. Make certain all restraining material (tape on legs, leg ropes, tail ropes, neck ropes, etc.) are removed. Do not remove the mouth rope or tape until last and ready to release.
5. The use of a wet cloth over the animal's eyes may make the release safer (especially with large animals).

6. After releasing the animal immediately leave the area. Do not poke, prod, or antagonize the animal in any way. Remaining restraint equipment such as a board or rope can be removed once the animals moves on its own. Check back regularly in order to remove equipment in a timely and safe manner.

7. Observe the animal to be sure that it is all right and does not over heat or chill. Normal basking and feeding behavior should commence within a few days. Contact Curator if not observed.

Vliet, K. A. Crocodilian Capture and Restraint. in *Zoo Animal and Wildlife Immobilization and Anesthesia* (eds. West, G., Heard, D. & Caulkett, N.) (John Wiley & Sons, Inc., 2014).
